# Physical Activity Protects the Pathological Alterations of Alzheimer’s Disease Kidneys via the Activation of PACAP and BMP Signaling Pathways

**DOI:** 10.3389/fncel.2020.00243

**Published:** 2020-08-14

**Authors:** Helga Perényi, Vince Szegeczki, Gabriella Horváth, Barbara Hinnah, Andrea Tamás, Zsolt Radák, Dóra Ábrahám, Róza Zákány, Dora Reglodi, Tamás Juhász

**Affiliations:** ^1^Department of Anatomy, Histology and Embryology, Faculty of Medicine, University of Debrecen, Debrecen, Hungary; ^2^Department of Anatomy, PTE-MTA PACAP Research Team, University of Pécs Medical School, Pécs, Hungary; ^3^Research Institute of Sport Science, University of Physical Education, Budapest, Hungary

**Keywords:** PACAP, Alzheimer’s disease, BMP signaling, physical activity, collagen type IV

## Abstract

Alzheimer’s disease (AD) is a neurodegenerative disorder with typical amyloid beta (Aβ) aggregations. Elimination of the Aβ precursors *via* the kidneys makes the organ a potential factor in the systemic degeneration leading to AD. Pituitary adenylate cyclase-activating polypeptide (PACAP) exerts neuroprotective effects in AD and plays a protective role in kidney pathologies. Increased physical activity is preventive of the formation of AD, but its detailed mechanism and possible connections with PACAP have not been clarified. In the kidneys of AD mice, the effects of physical activity were investigated by comparing wild-type and AD organs. Aβ plaque formation was reduced in AD kidneys after increased training (TAD). Mechanotransduction elevated PACAP receptor expression in TAD mice and normalized the protein kinase A (PKA)-mediated pathways. BMP4/BMPR1 elevation activated Smad1 expression and normalized collagen type IV in TAD animals. In conclusion, our data suggest that elevated physical activity can prevent the AD-induced pathological changes in the kidneys *via*, at least in part, the activation of PACAP–BMP signaling crosstalk.

## Introduction

Alzheimer’s disease (AD), a degenerative process in the central nervous system (CNS), is the most frequent cause of dementia in elderly patients, resulting in neuronal loss and leading to progressive cognitive deficit (Burns and Iliffe, 2009; Dong Y. et al., 2019; Nazarian et al., 2019; Cervellati et al., 2020; Pomilio et al., 2020). Because of its increased prevalence, AD is one of the major priorities of healthcare systems, and various attempts have been made in the last decades to find reliable prevention methods or novel therapeutic approaches (Belghali et al., 2017; Panda and Jhanji, 2019; Whitehouse, 2019). One of the first signs of AD formation and manifestation is the pathological aggregation of amyloid beta (Aβ) deposits and the abnormal accumulation of tau proteins followed by inflammation (Tiraboschi et al., 2004). Amyloidogenesis is a well-controlled process in healthy tissues, but pathologic amyloid plaques can accumulate in various tissues in Alzheimer’s disease (Whelly et al., 2012; Reglodi et al., 2018c). Accumulation of amyloid plaques has also been identified in peripheral organs, resulting in a systemic disorder (Morris et al., 2014; Reglodi et al., 2018c; Pomilio et al., 2020). Among others, Aβ aggregation pathologically alters pancreatic function (Kawarabayashi et al., 1996; Arbor et al., 2016; Tavares et al., 2017) and can negatively affect renal function (Kheirbakhsh et al., 2018).

Pituitary adenylate cyclase-activating polypeptide (PACAP) is a 38-amino acid C-terminally α-amidated peptide that was first isolated in 1989 from an ovine hypothalamic extract by Miyata and colleagues (Miyata et al., 1989). PACAP belongs to the vasoactive intestinal polypeptide (VIP)–secretin–growth hormone-releasing hormone (GHRH)–glucagon superfamily. PACAP has three major G protein-coupled seven-transmembrane receptors: PAC1, VPAC1, and VPAC2. PAC1 is responsible for specific PACAP binding, while the VPAC1 and VPAC2 receptors have equal affinity to bind PACAP and VIP (Laburthe et al., 2002). PACAP and its receptors have been detected in the CNS and many peripheral tissues such as the respiratory tract, urinary tract, and digestive system (Vaudry et al., 2009), or in bone and chondrogenic cultures (Juhasz et al., 2015a; Jozsa et al., 2018). The protective/preventive role of PACAP has been proven in several age-related disorders, and lack of PACAP leads to accelerated aging (Reglodi et al., 2018a; Szegeczki et al., 2019). PACAP has been shown to attenuate the severity of AD in different animal models and protects from Aβ toxicity (Han et al., 2014a; Maasz et al., 2017). We have previously described that PACAP-deficient mice are prone to develop a presenile systemic amyloidosis, with peripheral organs severely affected (Reglodi et al., 2018c). The most dramatic amyloid deposition was found in the kidneys of aging PACAP knockout mice, where 100% of the animals developed a severe glomerular infiltration causing functional failure (Gronewold et al., 2017; Reglodi et al., 2018c). In addition, PACAP has a nephroprotective role in various kidney pathologies, such as toxic nephropathies and ischemia/reperfusion-induced kidney injury of rats (Horvath et al., 2019; Laszlo et al., 2019). Binding to its receptors, PACAP initiates the activation of various signaling pathways *via* the activation of intracellular messengers such as adenylate cyclase, which triggers protein kinase A (PKA), and subsequent activation of the phosphorylate transcription factors such as CREB and mediates different gene expressions (Kienlen Campard et al., 1997; Juhasz et al., 2014b; Dong Q. et al., 2019). PACAP has a modulatory role in several signaling cascades such as Notch and sonic hedgehog signaling (Sandor et al., 2014; Fulop et al., 2019), Sox transcription factor activation (Szegeczki et al., 2019), and has an important balancing function in bone morphogenetic protein (BMP) signaling (Jozsa et al., 2019; Laszlo et al., 2019).

BMP is a member of the transforming growth factor beta (TGF-β) protein family predominantly detected in bone and has several functions, from the embryonic development to the homeostasis of adult tissues (Bandyopadhyay et al., 2013). BMPs can bind to serine/threonine protein kinase receptor (BMPR1), the downstream targets of which bind the corresponding DNA sequence through the Smad cascade and regulate the expression of certain genes, such as those coding for collagens (Von Bubnoff and Cho, 2001). BMP4 is one of the major cytokines which regulate proper kidney development (Nishinakamura and Sakaguchi, 2014; Mills et al., 2017). BMP receptor activation *via* BMP4 influences the expression of basement membrane components, such as collagen type IV (Matsubara et al., 2015; Laszlo et al., 2019), which also plays an important role in proper filtration. Alterations in BMP4 expression have also been demonstrated in AD (Li D. et al., 2008). BMP and PACAP signaling crosstalk has already been shown in various processes. PACAP has been demonstrated to play a role as an antagonist of BMP4 in *Xenopus* early development (Otto et al., 2000), and BMPs can modulate PACAP function in peptidergic system formation (Pavelock et al., 2007). The expression of BMPs is altered in bone development processes (Jozsa et al., 2018) and shows a shifted expression pattern in the callus formation of PACAP knockout (KO) mice (Jozsa et al., 2019). The addition of PACAP increased BMP4 expression in the UMR-106 osteogenic cell line (Juhasz et al., 2014b). Moreover, we have demonstrated that PACAP addition elevated BMP4 expression in ischemia-induced kidney injury (Laszlo et al., 2019).

For detailed investigation of AD formation, various *in vivo* and *in vitro* experiments have been performed (Wu et al., 2019) in order to follow the molecular biological alterations. There are a few genetically modified mice with tau and Aβ overexpression showing macroscopical and molecular biological disorders of AD (Radak et al., 2010; Abraham et al., 2019). In these models, it has been proven that physical activity was able to postpone the manifestation of AD, but its detailed mechanism has not yet been investigated (Radak et al., 2010). As PACAP has an important protective role in the kidneys and has a direct protective role in AD, it could be one of the target molecules to positively influence Aβ elimination *via* the kidneys (Leckstrom et al., 1997).

In our experiments, we present evidence that physical activity has direct effects on AD in the kidneys of Alzheimer’s disease mouse models. Furthermore, we show that PACAP and BMP signaling are also involved in the prevention of illness formation *via* physical activation.

## Materials and Methods

### Animals

Male Alzheimer transgenic [B6C3-Tg(APPswe,PSEN1dE9)85Dbo/J] (*n* = 5) mice were used to follow the effects of AD. Three-month-old wild type (WT; no transgenic modulation and no training, *n* = 5), Alzheimer transgenic mice (AD, *n* = 5), and trained Alzheimer’s disease mice (TAD, *n* = 5) were kept under light/dark cycles of 12:12 h with food and water *ad libitum*. Alzheimer transgenic mice were trained on a treadmill four times per week for 1 h divided into 10 sessions. One session contained 2 min of low-intensity running (10 m/min) and 4 min of high-intensity running (20 m/min). The study was carried out in accordance with ethical guidelines (ethical permission number: PEI/001/2105-6/2014, Semmelweis University, Hungary). Genotyping was performed using a Phire Animal Tissue Direct PCR Kit (Thermo Fischer Scientific, Waltham, MA, United States) according to the manufacturer’s instructions.

### Light Microscopical Morphology

Kidneys were washed in phosphate-buffered saline (PBS) three times and fixed in a 4:1 mixture of absolute ethanol and 40% formaldehyde, then embedded in paraffin. Serial sections were made and hematoxylin–eosin (HE) staining (Sigma-Aldrich, MO, United States) for morphological analysis and Congo red staining (Abcam, Cambridge, United Kingdom) for Aβ accumulation were performed. The staining protocols were carried out according to the manufacturer’s instructions. Photomicrographs were taken using a DP74 camera (Olympus Corporation, Tokyo, Japan) on an Olympus Bx53 microscope (Olympus Corporation, Tokyo, Japan). For measurement of the staining intensity, we used ImageJ 1.40g freeware. Pixel density analysis was performed from at least three photos of five independent experiments.

### Immunohistochemistry

Immunohistochemistry was performed on WT, AD, and TAD mice kidney tissue samples to visualize the localization of CREB, Smad1, and collagen type IV (Col. IV). The kidneys were fixed in a 4:1 mixture of absolute ethanol and 40% formaldehyde and washed in 70% ethanol. After embedding, serial sections were made and deparaffinization was then followed by rinsing in PBS (pH 7.4). Non-specific binding sites were blocked with PBS supplemented with 1% bovine serum albumin (Amresco LLC, Solon, OH, United States), then the samples were incubated with polyclonal CREB (Millipore, MO, United States) at a dilution of 1:600, Smad1 (Cell Signaling, Danvers, MA, United States) at a dilution of 1:500, or Col. IV (Abcam, Cambridge, United Kingdom) antibody at a dilution of 1:600 at 4°C overnight. For visualization of the primary antibodies, anti-rabbit Alexa Fluor 555 secondary antibody (Life Technologies Corporation, Carlsbad, CA, United States) was used at a dilution of 1:1,000. The samples were mounted in a Vectashield mounting medium (Vector Laboratories, Peterborough, United Kingdom) containing 4’,6-diamidino-2-phenylindole (DAPI) for nuclear DNA staining. For the negative controls, anti-rabbit Alexa Fluor 555 was used without the primary antibodies. For the detection of Col. IV, photomicrographs were taken using the DP74 camera (Olympus Corporation, Tokyo, Japan) on an Olympus Bx53 microscope (Olympus Corporation, Tokyo, Japan). Images were acquired using cellSense Entry 1.5 software (Olympus, Shinjuku, Tokyo, Japan) with constant camera settings to allow comparisons of the fluorescent signal intensities. For CREB and Smad1, an Olympus FV1000S confocal microscope (Olympus Co., Tokyo, Japan) was used applying × 60 oil immersion objective (NA: 1.3). For excitation, laser lines of 543 nm were used. The average pixel time was 4 μs. Z image series of 1 μm optical thickness were recorded in sequential scan mode. Images of Alexa555 and DAPI were overlaid using Adobe Photoshop version 10.0 software. Contrast of images was equally increased without changing constant settings.

### RT-PCR Analysis

The kidneys of WT (*n* = 5), AD (*n* = 5), and TAD (*n* = 5) mice were mechanically ground and were dissolved in Trizol (Applied Biosystems, Foster City, CA, United States), after 30 min incubation on 4°C, and total RNA was isolated. RNA was harvested in RNase-free water and stored at -70°C. Reverse transcription was performed by using High-Capacity RT kit (Applied Biosystems, Foster City, CA, United States). For the sequences of primer pairs and details of the polymerase chain reactions (see Table 1). Amplifications were performed in a thermal cycler (Labnet MultiGene 96-well Gradient Thermal Cycler; Labnet International, Edison, NJ, United States) as follows: 95°C, 2 min, followed by 35 cycles (denaturation, 94°C, 30 s; annealing for 45 s at optimized temperatures as given in Table 1; and extension, 72°C, 90 s) and then 72°C, 7 min. Actin was used as the internal control. PCR products were analyzed using a 1.2% agarose gel containing ethidium bromide. The optical densities of the PCR product signals were determined by using ImageJ 1.40g freeware.

**TABLE 1:** Nucleotide sequences, amplification sites, GenBank accession numbers, amplimer size, and PCR reaction conditions for each primer pair.

Gene	Primer	Nucleotide sequence (5′→3′)	Genbank ID	Annealing temperature (°C)	Amplimer size (bp)
PAC1 (ADCYAP1R1)	Sense	TATTACTACCTGTCGGTGAAG (912–932)	NM_007407.4	52	213
	Antisense	ATGACTGCTGTCCTGCTC (1107–1124)			
VPAC1 (VIPR1)	Sense	TTT GAG GAT TTC GGG TGC (974–991)	NM_011703.4	53	266
	Antisense	TGG GCC TTA AAG TTG TCG (1222–1239)			
VPAC2 (VIPR2)	Sense	CTC CTG GTA GCC ATC CTT (805–822)	NM_009511.2	53	149
	Antisense	ATG CTG TGG TCG TTT GTG (936–953)			
PKA (Prkaca)	Sense	GCAAAGGCTACAACAAGGC (847–865)	NM_008854	53	280
	Antisense	ATGGCAATCCAGTCAATCG (1109–1126)			
CREB (Creb1)	Sense	AGA TTG CCA CAT TAG CCC (95–112)	NM031017.1	52	441
	Antisense	GCT GTA TTG CTC CTC CCT (518–535)			
BMP2 (Bmp2)	Sense	AAG CCA GGT GTC TCC AAG (697–714)	NM_017178.1	53	209
	Antisense	AAG TCC ACA TAC AAA GGG TG (886–905)			
BMP4 (Bmp4)	Sense	TAG TCC CAA GCA TCA CCC (876–893)	NM_012827.2	53	294
	Antisense	TCG TAC TCG TCC AGA TAC AAC (1149–1169)			
BMPR1 (Bmpr1a)	Sense	CCA TTG CTT TGC CAT TAT (240–257)	NM_009758.4	47	487
	Antisense	TTT ACC AAC CTG CCG AAC (709–726)			
Smad1 (Smad1)	Sense	AGC ACC TAC CCT CAC TCC C (935–953)	NM_013130.2	56	306
	Antisense	GAA ACC ATC CAC CAA CAC G (1222–1240)			
Collagen type IV (Col4a1)	Sense	TCG GCT ATT CCT TCG TGA TG (4963–4982)	NM_007735.2	56	209
	Antisense	GGA TGG CGT GGG CTT CTT (5154–5171)			
Actin (Actb)	Sense	GCCAACCGTGAAAAGATGA (419–437)	NM_007393.5	54	462
	Antisense	CAAGAAGGAAGGCTGGAAAA (861–880)			

### Western Blot Analysis

The kidneys of WT (*n* = 5), AD (*n* = 5), and TAD (*n* = 5) mice were washed in physiological saline and stored at -70°C. The samples were mechanically disintegrated with a tissue grinder in liquid nitrogen. Then, they were collected in 100 μl of homogenization RIPA (radioimmunoprecipitation assay) buffer (150 mM sodium chloride, 1.0% NP40, 0.5% sodium deoxycholate, 50 mM Tris, pH 8.0) containing protease inhibitors (10 μg/ml aprotinin, 5 mM benzamidine, 10 μg/ml leupeptin, 10 μg/ml trypsin inhibitor, 1 mM PMSF, 5 mM EDTA, 1 mM EGTA, 8 mM Na-fluoride, and 1 mM Na-orthovanadate). The suspensions were sonicated by pulsing burst for 30 s at 40 A (Cole-Parmer, IL, United States). The total cell lysates for Western blot analyses were prepared. Forty micrograms protein was separated in 7.5% SDS–polyacrylamide gels for the detection of PAC1, VPAC1, VPAC2, PKA, P-PKA, CREB, P-CREB, BMP2, BMP4, BMPR1, Smad1, Col. IV, and actin. The proteins were transferred electrophoretically to nitrocellulose membranes and exposed to the primary antibodies overnight at 4°C in the dilution, as given in Table 2. After washing for 30 min with PBS Tween (PBST), the membranes were incubated with the peroxidase-conjugated secondary antibody anti-rabbit IgG in a 1:1,500 (Bio-Rad Laboratories, CA, United States) or anti-mouse IgG in a 1:1,500 (Bio-Rad Laboratories, CA, United States) dilution. The signals were detected with enhanced chemiluminescence (Advansta Inc., Menlo Park, CA, United States) according to the instructions of the manufacturer. Actin was used as an internal control. The signals were developed with a gel documentary system (Fluorchem E, ProteinSimple, CA, United States). The optical densities of the signals were measured by using ImageJ 1.40g freeware.

**TABLE 2:** Antibodies used in the experiments.

Antibody	Host animal	Dilution	Distributor
Anti-BMP4	Rabbit, polyclonal	1:600	Cell Signaling, Danvers, MA, United States
Anti-BMPR1	Mouse, monoclonal	1:600	Abcam, Cambridge, United Kingdom
Anti-Smad1	Rabbit, polyclonal	1:600	Cell Signaling, Danvers, MA, United States
Anti-CREB	Rabbit, polyclonal	1:800	Millipore, Billerica, MA, United States
Anti-P-CREB	Rabbit, polyclonal	1:800	Millipore, Billerica, MA, United States
Anti-Col. IV	Mouse, monoclonal	1:500	Abcam, Cambridge, United Kingdom
Anti-PKA	Rabbit, polyclonal	1:800	Cell Signaling, Danvers, MA, United States
Anti-P-PKA	Rabbit, polyclonal	1:600	Cell Signaling, Danvers, MA, United States
Anti-BMP2	Mouse, monoclonal	1:500	Abcam, Cambridge, United Kingdom
Anti-actin	Mouse, monoclonal	1:10,000	Sigma-Aldrich, St. Louis, MO, United States

### Statistical Analysis

All data are representative of at least five independent experiments. For all figures, the samples of the same WT, AD, and TAD animals were chosen with their inner control for a better comparison. Changes were based on the five results, but only one demonstrative photo from the same animal group was used in every figure. Statistical analysis was performed by ANOVA and unpaired Student’s *t*-test. The threshold for the statistically significant differences as compared to the control samples was set at ^∗^*p* < 0.05 and to the AD samples at ^#^*p* < 0.05.

## Results

### Reduced Aβ Accumulation After Physical Activity in AD Kidneys

**FIGURE 1:**
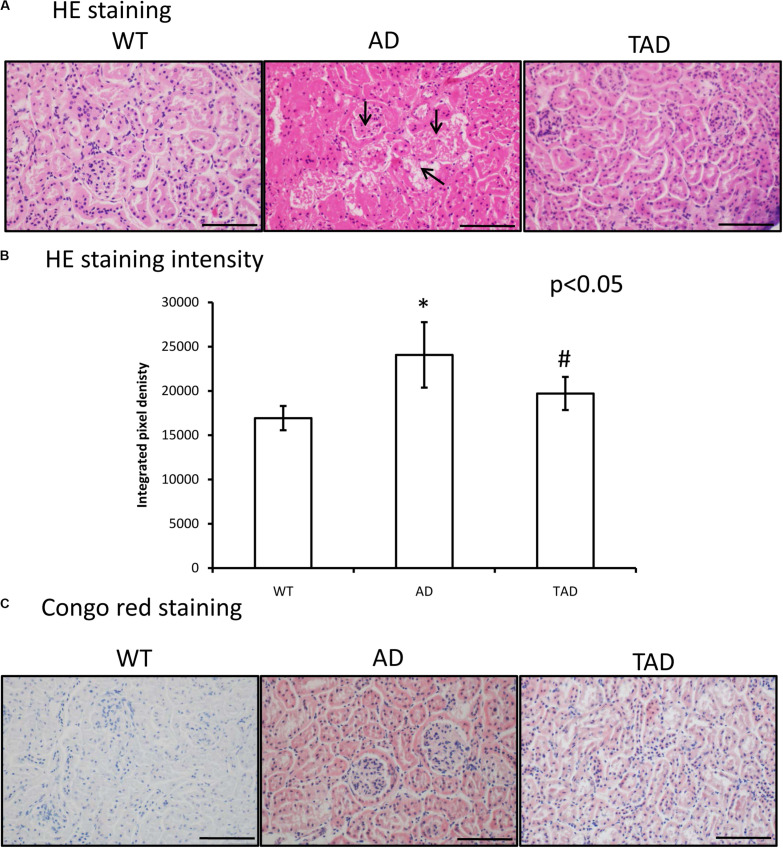
**(A)** Representative microphotograph of wild-type (WT), Alzheimer’s disease (AD), and trained Alzheimer’s disease (TAD) kidneys stained with hematoxylin and eosin. Overview of the kidney cortex. Original magnification was × 20. *Scale bar*, 50 μm. *Arrows* show a representative area for amyloid beta (Aβ) plaque formation. **(B)** Staining intensity analysis. Representative data of five independent experiments. The staining intensities are presented as bar graphs ± SEM. *Asterisks* indicate significant (^∗^*p* < 0.05) differences in the staining pixel intensity compared to WT and (^#^*p* < 0.05) compared to AD. **(C)** Representative microphotographs of WT, AD, and TAD kidneys stained with Congo red. *Red colors* show Aβ accumulation. Original magnification was × 20. *Scale bar*, 50 μm.

In WT mice, a normal kidney cortex morphology was demonstrated (Figure 1A), without any Congo red positivity (Figure 1C). On the contrary, Aβ plaque accumulation was detectable in AD mice (Figure 1A). Homogenous eosinophilic deposits were visible, which are typical features of Aβ appearance in the interstitium of AD kidneys (Figure 1A). Strong Aβ positivity could be seen in the proximal and distal tubules and the Bowman capsule of AD kidneys with Congo red staining (Figure 1C). Moreover, the tubular systems of AD kidneys showed a strong eosinophilic appearance. Pixel analysis revealed a significantly elevated staining intensity in AD kidneys compared with the WT kidneys (Figure 1B). Interestingly, increased physical activity diminished the eosinophilic deposits in the interstitium and also reduced Aβ accumulation in the cortical tubules (Figures 1A,C, respectively). Physical activity decreased the elevated staining intensity in TAD animals compared with the AD samples (Figures 1A,B).

### Normalized Receptors of PACAP Signaling in TAD Animals

**FIGURE 2:**
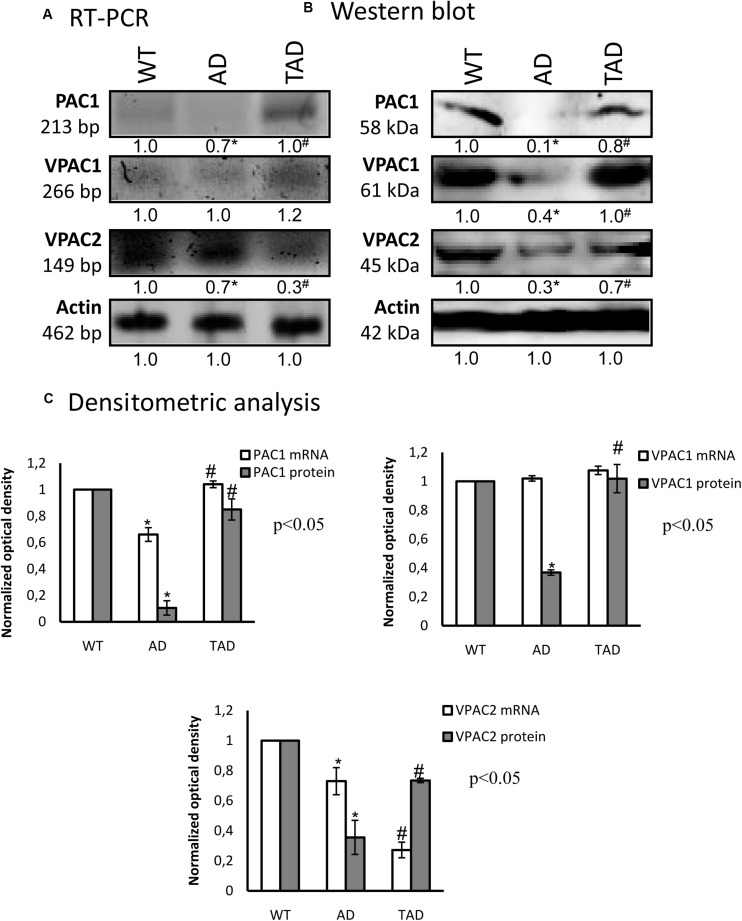
mRNA **(A)** and protein **(B)** expressions of pituitary adenylate cyclase-activating polypeptide (PACAP) receptors in the kidneys. The optical density of the signals was measured and the results were normalized to the optical density of the wild type (WT). For **(A,B)**, the *numbers below the signals* represent the integrated densities of the signals determined by ImageJ software. *Asterisks* indicate significant (^∗^*p* < 0.05) alterations of expressions as compared to the WT and (^#^*p* < 0.05) compared to Alzheimer’s disease (AD). Representative data of five independent experiments. For reverse transcription PCR (RT-PCR) and for Western blot, actin was used as the control. **(C)** Statistical analysis of the RT-PCR and Western blot data. All data presented are the averages of at least five different experiments. Statistical analysis was performed with Student’s *t-*test. All data were normalized on actin and are expressed as the mean ± SEM.

In WT kidneys, the messenger RNAs (mRNAs) of all PACAP receptors were detectable (Figure 2A). Some alterations were shown with the RT-PCR reactions in AD mice, which were normalized in TAD animals (Figures 2A,C). The protein expressions of PAC1, VPAC1, and VPAC2 receptors were equally demonstrated in WT kidneys (Figures 2B,C). On the contrary, PAC1 receptor protein expression was almost undetectable, and strongly reduced VPAC1 and VPAC2 protein expressions were demonstrated in AD kidneys (Figures 2B,C). Physical activity increased the expressions of PACAP receptors in TAD kidneys (Figures 2B,C). Most dominantly, the protein expressions of PAC1 and VPAC1 receptors were elevated (Figures 2B,C).

### Altered Canonical Signaling Pathways of PACAP in AD

**FIGURE 3:**
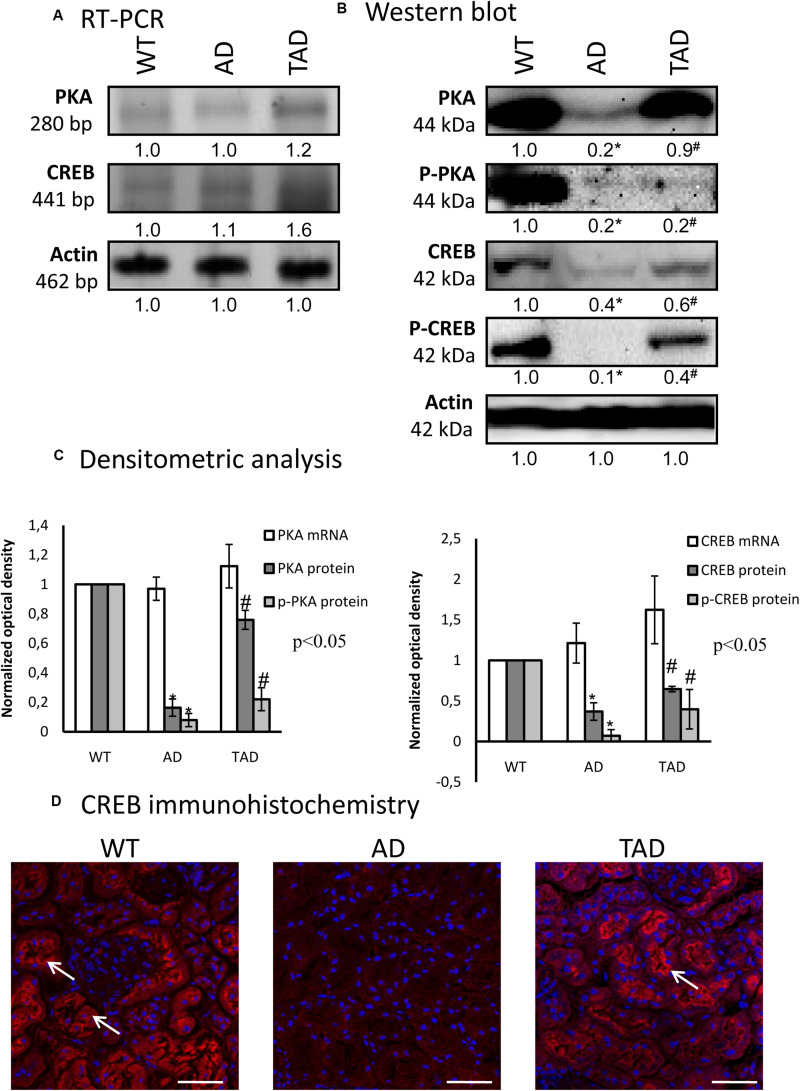
mRNA **(A)** and protein **(B)** expressions of pituitary adenylate cyclase-activating polypeptide (PACAP) signaling in the kidneys. The optical density of the signals was measured and the results were normalized to the optical density of the controls. For **(A,B)**, the *numbers below the signals* represent the integrated densities of the signals determined by ImageJ software. *Asterisks* indicate significant (^∗^*p* < 0.05) alterations of expressions as compared to the wild type (WT) and (^#^*p* < 0.05) compared to Alzheimer’s disease (AD). Representative data of five independent experiments. For reverse transcription PCR (RT-PCR) and for Western blot, actin was used as the control. **(C)** Statistical analysis of the RT-PCR and Western blot data. All data presented are the averages of at least five different experiments. Statistical analysis was performed with Student’s *t*-test. All data were normalized on actin and are expressed as the mean ± SEM. **(D)** Immunohistochemistry of CREB in the cortex of the kidneys. *Arrows* show the apical part of the proximal tubules. Magnification was made with × 60 objective. *Scale bar*, 20 μm.

To demonstrate the involvement of PACAP signaling in AD kidneys, first, we investigated the canonical downstream targets. PKA is activated by the elevated adenylate cyclase activity; subsequently, the phosphorylation of PKA is increased and triggers the transcriptional activity of CREB. In all experimental groups, the mRNAs of PKA and CREB were demonstrated without significant alterations (Figures 3A,C). In AD kidneys, a diminished PKA protein expression was detected, which was increased in TAD animals (Figures 3B,C). In TAD kidneys, the PKA expression almost normalized and reached the basal level, but the more active phosphorylated form of PKA after a strong reduction in AD mice did not re-increase (Figures 3B,C). Similarly, CREB protein expression was reduced in AD kidneys; moreover, its phosphorylated, thus more active, form was undetectable in AD (Figures 3B,C). The increased activity of AD mice augmented the expression of CREB and elevated the phosphorylation level of the transcription factor (Figures 3B,C). CREB expression and specific localization in proximal tubules have been demonstrated earlier (Taub, 2018). In WT kidneys, CREB appears in the cytoplasm and shows an apical accumulation in proximal tubules; only cytoplasmic signals appeared in distal tubules and barely detectable CREB was visible in the Bowman capsule (Figure 3D). CREB signal diminished and no subcellular specificity could be analyzed with this method in AD kidneys (Figure 3D). Interestingly, strong apical signals were demonstrated in proximal tubular cells of TAD kidneys, but no further alterations were shown in distal tubules and in renal corpuscles (Figure 3D).

### Modified BMP Signaling in AD

**FIGURE 4:**
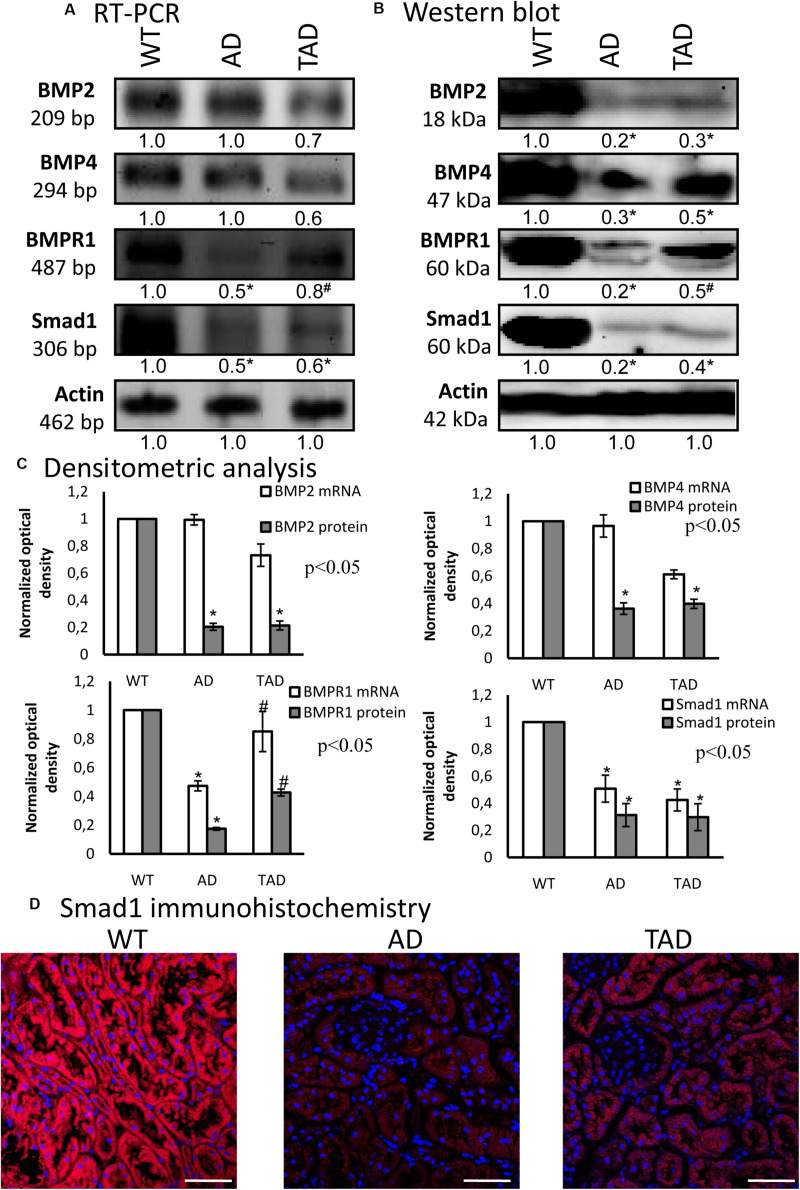
mRNA **(A)** and protein **(B)** expressions of bone morphogenetic protein (BMP) signaling in the kidneys. For reverse transcription PCR (RT-PCR) and for Western blot, actin was used as the control. The optical density of the signals was measured and the results were normalized to the optical density of the controls. For panels **(A)** and **(B)**, the *numbers below the signals* represent the integrated densities of the signals determined by ImageJ software. *Asterisks* indicate significant (^∗^*p* < 0.05) alterations of expressions as compared to the wild type (WT) and (^#^*p* < 0.05) compared to Alzheimer’s disease (AD). Representative data of five independent experiments. **(C)** Statistical analysis of the RT-PCR and Western blot data. All data presented are the averages of at least five different experiments. Statistical analysis was performed with Student’s *t*-test. All data were normalized on actin and are expressed as the mean ± SEM. **(D)** Immunohistochemistry of Smad1 in the cortex of the kidneys. Magnification was made with × 60 objective. *Scale bar*, 20 μm.

For the investigation of PACAP signaling crosstalk, we followed the BMP signaling pathways. First, the mRNAs of BMP2 and BMP4 were detected in all experimental groups without significant alterations (Figures 4A,C). BMPR1 and Smad1 mRNA expressions were reduced in AD kidneys, but elevated in TAD kidneys (Figures 4A,C). The protein expression of BMP2 diminished in AD kidneys and did not increase in TAD animals (Figures 4B,C). Furthermore, AD kidneys showed a decreased BMP4 expression, but it was augmented in TAD animals (Figures 4B,C). A similar pattern was demonstrated in the expression of BMPR1, which normalized in TAD kidneys after a strong AD reduction (Figures 4B,C). The protein expression of Smad1 is strongly reduced in AD kidneys, and a lower but significant elevation was detected in TAD animals (Figures 4B,C). With immunohistochemistry, a strong cytoplasmic signal of Smad1 was demonstrated in WT kidneys (Figure 4D). In AD animals, Smad1 reduction was visible in the tubular system, but it increased in the proximal tubules of TAD kidneys (Figure 4D).

### Altered Expressions of Basement Membrane Components in AD

**FIGURE 5:**
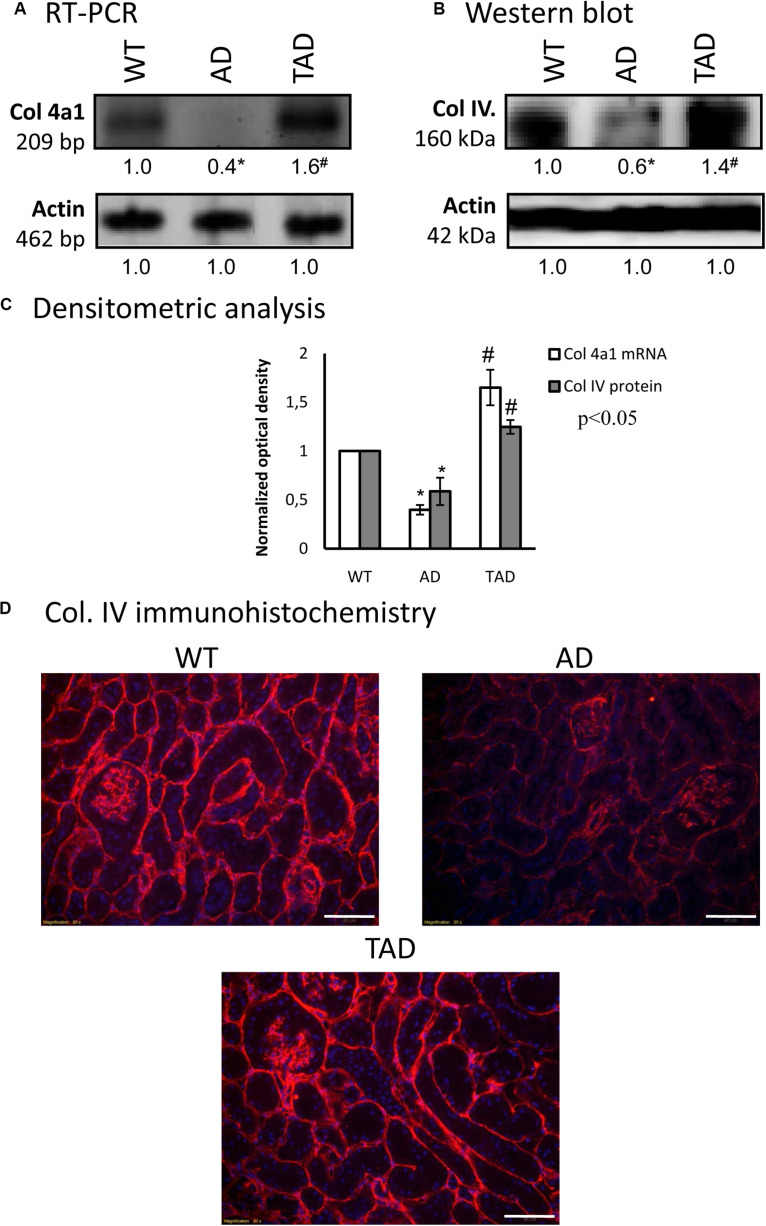
mRNA **(A)** and protein **(B)** expressions of collagen type IV in the kidneys. For reverse transcription (RT-PCR) and for Western blot, actin was used as the control. The optical density of the signals was measured and the results were normalized to the optical density of the controls. For **(A,B)**, the *numbers below the signals* represent the integrated densities of the signals determined by ImageJ software. *Asterisks* indicate significant (^∗^*p* < 0.05) alterations of expressions as compared to the wild type (WT) and (^#^*p* < 0.05) compared to Alzheimer’s disease (AD). Representative data of five independent experiments. **(C)** Statistical analysis of the RT-PCR and Western blot data. All data presented are the averages of at least five different experiments. Statistical analysis was performed with Student’s *t*-test. All data were normalized on actin and are expressed as the mean ± SEM. **(D)** Immunohistochemistry of collagen type IV in the cortex of the kidneys. Magnification was made with × 20 objective. *Scale bar*, 50 μm.

As we have published earlier, the common target of BMP and PACAP signaling can be collagen type IV, which was also altered in ischemic conditions of the kidneys (Laszlo et al., 2019). The mRNA expression of collagen type IV was undetectable in AD kidneys, while it was demonstrated in TAD kidneys (Figures 5A,C). The protein expression of this collagen showed a prominent reduction in AD kidneys, but it was significantly elevated in TAD animals (Figures 5B,C). On the other hand, the immunopositivity of collagen type IV was decreased dominantly around the tubules of AD kidneys, while no such strong reduction was detected around the renal corpuscles (Figure 5D). In TAD animals, the signals of collagen type IV were elevated around the tubules and renal corpuscles (Figure 5D).

## Discussion

Numerous molecular and neuropathological disorders have been described in AD, such as tau hyperphosphorylation (Sierra-Fonseca and Gosselink, 2018), modification of Ca^2+^ signaling pathways (Glaser et al., 2019), and Aβ accumulation (Naseri et al., 2019), leading to neuronal and synaptic disorders (Cardozo et al., 2019). In Alzheimer’s disease, several peripheral organs are also involved, such as the testis (Tavares et al., 2017), pancreas (Kulas et al., 2017), and kidneys (Paterson et al., 2017), indicating a complex systemic disease (Morris et al., 2014). Pathological Aβ accumulation in the kidneys (Gronewold et al., 2017) may result in filtration disorder. Although various signaling molecules have been identified as possible targets in the treatment of this neurodegeneration process (Loera-Valencia et al., 2019; Patel et al., 2019), the complexity of AD makes drug development difficult. Limited data show the importance of physical activity in postponing the manifestation of characteristic AD (Radak et al., 2010; Abraham et al., 2019). One of the possible protective agents could be PACAP, which has been described to have a preventive effect on AD formation (Han et al., 2014b). PACAP has also been shown to modify amyloid aggregation (Reglodi et al., 2018c) and has important preventive functions in aging (Reglodi et al., 2018a; Szegeczki et al., 2019). The general protective role of the neuropeptide has been demonstrated in various disorders, such as retinopathy (Vaczy et al., 2018), diabetes (Banki et al., 2014), Parkinson’s disease (Reglodi et al., 2006; Reglodi et al., 2017), and inflammatory processes (Sakamoto et al., 2015). It has been shown that PACAP diminishes the harmful effects of oxidative stress (Juhasz et al., 2014a), ischemic conditions (Reglodi et al., 2018d), and mechanical overload (Juhasz et al., 2015b). Although the positive function of PACAP in AD has been published, a detailed analysis in peripheral organs has still not been completely performed.

In an AD mouse model system, we investigated the kidneys and the effects of physical exercise in connection to PACAP signaling pathways. Similarly to previous results, we identified a strong Aβ accumulation and intensive eosinophilia in AD kidneys (Gronewold et al., 2017). In contrast, the microscopical signs of Aβ deposits were not detectable after physical activity. Although cortical accumulation of Aβ has not been altered in high-intensity training (Frederiksen et al., 2019), it has also been published that training and running have a protective effect in the hippocampus of mice in AD (Schimidt et al., 2019). Although physical activity generally decreases the amount of Aβ deposits or concentration in the blood and cerebrospinal fluid (Brown et al., 2019), no data are available to clarify the mechanism in the periphery. We can conclude that elevated physical activity protects the kidneys from Aβ accumulation. We have demonstrated that PACAP has a direct connection with mechanotransduction (Juhasz et al., 2015b; Szentleleky et al., 2019). Therefore, we investigated the possible alterations of the expressions of PACAP receptors in the kidneys of AD and trained AD animals. The expressions of all PACAP receptors were demonstrated in the kidneys, with a significant decrease in the AD samples. The protein expressions of all PACAP receptors recovered in TAD animals. VPAC1 has already been shown to be expressed in the highest level in proximal tubules (Eneman et al., 2016), and the expression of the PAC1 receptor was demonstrated in the HK-2 cell line (Li et al., 2010). Direct binding of PACAP in kidney diseases has been proven both on PAC1 and VPAC1 receptors (Li M. et al., 2008), suggesting their equal importance in AD-related disorders. Reduction of the PAC1 receptors has been demonstrated in the CNS of the AD models (Han et al., 2014a), and mechanical stimulation increased the expression in chondrogenic cells (Juhasz et al., 2015b). The importance of the PACAP signaling pathway is also hallmarked by the elevated expression of the VPAC receptor in TAD, demonstrating the VIP/PACAP-related signaling crosstalk in AD (Dogrukol-Ak et al., 2004). Subsequently, we conclude that training and increased physical activity has a direct effect on AD *via* the activation of PACAP signaling. The most described PACAP-mediated signaling pathway is regulated by PKA (Vaudry et al., 2009), and its activity is reduced in AD (Kumar and Singh, 2018). Moreover, it has been published that the increased activity of PKA *via* CREB phosphorylation can regulate and suppress Aβ secretion in platelets (Sepulveda et al., 2019). We also detected PKA and CREB expression reductions in AD kidneys, while in TAD kidneys their expressions normalized. On the other hand, the autophosphorylated PKA is not affected by physical activity, suggesting a PACAP-independent regulation of PKA phosphorylation (Smith and Scott, 2018). Autophosphorylation of PKA is not necessary for the phosphorylation of CREB and its activity is not in ratio with its phosphorylation, but other signaling elements can be partly involved in the increased CREB phosphorylation, such as ERK activation (Racz et al., 2006). CREB is one of the transcription factors which can be phosphorylated by PKA (Zakany et al., 2002), and its activation in synapses is important in memory-related gene expression (Teich et al., 2015). CREB phosphorylation can be harmed in AD and result in alterations of synaptic plasticity (Teich et al., 2015). CREB is an essential factor in the regulation of the Na-K ATPase function in kidney proximal tubules (Taub, 2018); subsequently, it regulates their filtration function. Elevated physical exercise increased the expression and phosphorylation of this transcription factor in AD kidneys. Notably, CREB localization was detected in the apical part of the proximal tubular cells in WT animals. This apical accumulation diminished in AD, but was expressed in the proximal tubular cells of the TAD groups. This phenomenon further strengthened the hypothesis that CREB regulates proximal tubule function and physical activity may rescue the normal filtration in AD. These facts suggest that PKA activation *via* PACAP signaling is one of the molecular pathways involved in the physical activity-mediated protection mechanisms in AD.

PACAP signaling has various molecular crosstalks (Juhasz et al., 2015a), such as BMP signaling (Jozsa et al., 2018), sonic hedgehog signaling (Juhasz et al., 2015b), or Notch signaling (Fulop et al., 2019). The connection of PACAP signaling and BMPs has been shown in bone formation (Juhasz et al., 2014b; Jozsa et al., 2018, 2019) and cartilage development (Juhasz et al., 2014a). Moreover, PACAP has an effect on BMP signaling in the kidneys in ischemic–reperfusion conditions (Laszlo et al., 2019). It has also been shown that BMP expression and function can be modified in AD in glial cell differentiation (Kwak et al., 2014). In this neurodegeneration, Aβ can modify the BMP2 and BMP4 release (Kwak et al., 2014) and has an effect on other BMPs, such as increased BMP6 expression (Crews et al., 2010). BMP2 and BMP4 mRNA expressions were not altered. We demonstrated similar results in the femurs (Jozsa et al., 2018), testis (Reglodi et al., 2018b), and various other organs of PACAP KO mice (Reglodi et al., 2018c), where the Western blot and mRNA levels were not in correlation. In the UMR-106 cell line, PACAP did not alter the mRNA expression, but the protein expression was changed (Juhasz et al., 2014b). In high-density cell cultures, the addition of PACAP did not significantly modulate the mRNA expressions of certain matrix-producing enzymes or matrix proteins (Juhasz et al., 2014a, 2015b). These findings indicate that PACAP signaling exerts more pronounced effects on posttranscriptional events and cellular protein metabolism, and it has less influence on the gene expression of the targeted downstream molecules. Although the BMP2 protein expression was not altered in TAD animals, we detected an elevation in the BMP4 protein expression after training. This suggests that physical activity induces the activation of BMP signaling partly *via* PACAP receptor activation. BMPR1 induces the activation of Smad transcription factors and can regulate the expression of various genes, such as basement membrane components (Villacorte et al., 2016). Interestingly, the expression of BMPR1 was strongly decreased in AD kidneys, but recovered in TAD animals. The ratio of Smad1 reduction and its elevation was slightly lower than that of BMPR1, but the immunopositivity of elevated Smad1 in TAD kidneys was visible in the proximal tubules. It strongly suggests a direct communication between PACAP and BMP signaling in AD kidneys. We conclude that physical activity mediates the PACAP–BMP4 signaling axis to protect the function of kidneys in AD.

We have already demonstrated that BMP signaling activated the expression of collagen type IV in ischemic reperfusion injury (Laszlo et al., 2019). It has also been discussed that PACAP has an effect on basement membrane expression, especially collagen type IV, in the testes of PACAP KO mice (Reglodi et al., 2018b). Furthermore, PACAP was able to elevate collagen secretion in the cartilage (Juhasz et al., 2014a). Components of the basement membrane have been altered around the microvessels of the cortex in AD (Christov et al., 2008); subsequently, the blood–brain barrier can be compromised. After physical activity, we detected an elevated collagen type IV expression in AD animals. Collagen type IV has been shown to inhibit Aβ plaque formation (Kiuchi et al., 2002) and normalize the function of the blood–brain barrier. Therefore, the elevated collagen type IV expression in TAD kidneys indicates a normalized basement membrane formation, which can play a role in Aβ elimination in the kidneys.

## Conclusion

In conclusion, our data suggest that physical activity in AD can decrease the Aβ plaque formation and that elevated physical exercise affects the PACAP/BMP-mediated signaling pathway and CREB activation in the kidneys. The latter can regulate the Na-K ATPase in proximal tubules and the expression of collagen type IV, which in turn normalize the kidney tubular system function. Therefore, the systemic effect of physical activity can postpone the formation of AD *via* normalization of amyloid elimination *via* the kidneys.

## Data Availability Statement

All datasets generated for this study are included in the article/supplementary material, further inquiries can be directed to the corresponding author.

## Ethics Statement

The animal study was reviewed and approved by PEI/001/2105-6/2014, Semmelweis University, Hungary, 1091 Budapest, Üllõi út 93. fsz. 2.

## Author Contributions

TJ, DR, and GH contributed to the study conception and design. ZR and DÁ established the physical activity model, animal care, and operations were established. HP, VS, BH, and TJ performed the molecular biological analysis. BH, HP, and VS performed immunohistochemistry and histology. VS and TJ performed the statistical analysis. HP, BH, VS, and AT did the acquisition of data. TJ, AT, RZ, and DR analyzed and interpreted the data. HP, RZ, TJ, GH, and DR participated in drafting the manuscript. All authors contributed to the article and approved the submitted version.

## Conflict of Interest

The authors declare that the research was conducted in the absence of any commercial or financial relationships that could be construed as a potential conflict of interest.

## Abbreviations

Aβ: amyloid beta
AD mouse: Alzheimer’s disease mouse
APP: amyloid precursor protein
BMP: bone morphogenetic protein
cAMP: cyclic adenosine monophosphate
CNS: central nervous system
CREB: cAMP response element-binding protein
HE staining: hematoxylin and eosin staining
KO mouse: knockout mouse
mRNA: messenger ribonucleic acid
PAC1: pituitary adenylate cyclase-activating polypeptide type I receptor
PACAP: pituitary adenylate cyclase activating polypeptide
PKA: protein kinase A
P-PKA: phosphorylated protein kinase A
Smad: SMA (“small” worm phenotype) and *Drosophila* MAD (“Mothers Against Decapentaplegic”) TAD mouse, trained Alzheimer’s disease mouse
VPAC: vasoactive intestinal polypeptide receptor
WT mouse: wild-type mouse.
